# Rosiglitazone Treatment of Type 2 Diabetic *db*/*db* Mice Attenuates Urinary Albumin and Angiotensin Converting Enzyme 2 Excretion

**DOI:** 10.1371/journal.pone.0062833

**Published:** 2013-04-30

**Authors:** Harshita Chodavarapu, Nadja Grobe, Hari K. Somineni, Esam S. B. Salem, Malav Madhu, Khalid M. Elased

**Affiliations:** Department of Pharmacology and Toxicology, Boonshoft School of Medicine, Wright State University, Dayton, Ohio, United States of America; Max-Delbrück Center for Molecular Medicine (MDC), Germany

## Abstract

Alterations within the renal renin angiotensin system play a pivotal role in the development and progression of cardiovascular and renal disease. Angiotensin converting enzyme 2 (ACE2) is highly expressed in renal tubules and has been shown to be renoprotective in diabetes. The protease, a disintegrin and metalloprotease (ADAM) 17, is involved in the ectodomain shedding of several transmembrane proteins including ACE2. Renal ACE2 and ADAM17 were significantly increased in *db*/*db* mice compared to controls. We investigated the effect of the insulin sensitizer, rosiglitazone, on albuminuria, renal ADAM17 protein expression and ACE2 shedding in *db*/*db* diabetic mice. Rosiglitazone treatment of *db*/*db* mice normalized hyperglycemia, attenuated renal injury and decreased urinary ACE2 and renal ADAM17 protein expression. Urinary excreted ACE2 is enzymatically active. Western blot analysis of urinary ACE2 demonstrated two prominent immunoreactive bands at approximately 70 & 90 kDa. The predominant immunoreactive band is approximately 20 kDa shorter than the one demonstrated for kidney lysate, indicating possible ectodomain shedding of active renal ACE2 in the urine. Therefore, it is tempting to speculate that renoprotection of rosiglitazone could be partially mediated via downregulation of renal ADAM17 and ACE2 shedding. In addition, there was a positive correlation between blood glucose, urinary albumin, plasma glucagon, and triglyceride levels with urinary ACE2 excretion. In conclusion, urinary ACE2 could be used as a sensitive biomarker of diabetic nephropathy and for monitoring the effectiveness of renoprotective medication.

## Introduction

The prevalence of type 2 diabetes and the incidence of related complications like diabetic nephropathy have increased dramatically worldwide. This pandemic of diabetes is expected to escalate further as the population ages and obesity rates continue to soar. As the most common medical complications of diabetes include a range of progressive, chronic, renal, and cardiovascular problems, there is a great need for clinical tools that would enable the prevention and early diagnosis of such complications. Diabetic nephropathy is one of the major microvascular complications of type 2 diabetes, and a leading cause of end-stage renal disease indicating a failure of current available preventive therapeutic strategies. Microalbuminuria, defined as a urinary albumin excretion between 30 and 300 mg/day, is a commonly considered clinical sign of renal dysfunction and an early predictor of diabetic nephropathy [1]. Despite therapeutic intervention, kidney function progressively worsens in many diabetic patients underscoring the importance of novel and disease-specific biomarkers. The renal renin angiotensin system (RAS), specifically angiotensin II (Ang II), plays a pivotal role in the pathogenesis of diabetic nephropathy [2]. Ang II and angiotensin converting enzyme (ACE) are activated in type 2 diabetes [3], and are involved in the development of microvascular and macrovascular complications of diabetes such as nephropathy, retinopathy and cardiovascular disease [4]. In addition to glycemia control, lowering blood pressure as well as therapeutic approaches aimed at blocking the RAS, are strategies known to improve symptoms of diabetic nephropathy and preserve renal function in individuals with chronic disease due to different causes [5].

Angiotensin converting enzyme 2 (ACE2), a new enzyme within the RAS, is a type I transmembrane glycoprotein, which cleaves the C-terminal amino acid of Ang II to form Ang-(1–7) [6], [7]. ACE2 plays an integral role in the protection against renal damage and cardiovascular disease [8]–[10]. Administration of ACE2 inhibitor (MLN-4760) increases albuminuria, mesangial pathologies and fibronectin deposition in diabetic mice [11], [12]. ACE2 delivery in form of recombinant protein or virus ameliorates the progression of diabetes-related complications, such as nephropathy and retinopathy [13]–[15]. In addition, urinary ACE2 is elevated in patients with diabetic nephropathy, renal disease, or renal transplant suggesting a possible role of urinary ACE2 as a non-invasive disease biomarker [16]–[18]. A disintegrin and metalloprotease (ADAM) 17, also known as tumor necrosis factor α-converting enzyme (TACE), is involved in the ectodomain shedding of several membrane bound proteins [19]–[21]. This includes ADAM17-mediated ectodomain shedding of ACE2 in human embryonic kidney cells and airway epithelial cells [22], [23]. Loss of tissue inhibitor of metalloproteinase 3 (TIMP3), an endogenous inhibitor of ADAM17, has been shown to exacerbate diabetic nephropathy [24].

Accumulating evidence suggests that thiazolidinediones (TZDs) exhibit renoprotective effects. TZDs are synthetic ligands with high affinity toward the γ isoform of peroxisome proliferator activated receptor (PPARγ). TZDs used to be among the first line choice medications to control hyperglycemia in type 2 diabetic patients [25]. Although TZDs are very effective in glycemic control, meta-analysis suggests that they can also increase the risk of macrovascular complications [26]. For this reason, the use of TZDs is restricted in the United States in favor of less aggressive treatment options. Clinical studies revealed that chronic treatment with the TZD rosiglitazone markedly reduced urinary albumin excretion in type 2 diabetic patients [27], [28]. In addition, TZDs modulate the RAS, in particular ACE [29], Ang II receptors [30], Ang I and Ang II levels [31]. However, the effect of TZD’s on ACE2 has not been investigated before.Our central hypothesis is that renal ACE2 is upregulated to combat the detrimental effects of enhanced Ang II during early stages of diabetic kidney damage. Ang II is known to activate and redistribute ADAM17 [32], causing ACE2 to be shed from the renal tubules into urine. This loss of renal ACE2 function leads to increased renal injury, which can be reversed by anti-diabetic medication with rosiglitazone resulting in reduced renal ADAM17 expression and consequently, decreased urinary ACE2 levels and improved renal ACE2 function.

## Materials and Methods

### Animals

Male *db/db* diabetic mice [33] with background strain C57BL/KsJ (BKS.Cg-Dock7*m* +/+ Lepr*^db^*/J) and their age-matched non-diabetic lean control mice (*db/m*) were obtained from Jackson Laboratories (Bar Harbor, ME, USA). Mice were housed individually with free access to food and water on a 12 hour light/dark cycle.

### Study Design

Six week old mice were randomly assigned to four treatment groups: 1) control group fed normal chow, 2) control group fed rosiglitazone diet (20 mg/kg/day; LKT laboratories, MN, USA), 3) *db/db* group fed normal chow and 4) *db/db* group fed rosiglitazone diet (20 mg/kg/day). Mice were monitored weekly for blood glucose, body weight, food intake, water intake, and urine output over a period of eight weeks. Body composition was measured in conscious mice using ^1^H magnetic resonance spectroscopy (EchoMRI-100, Echo Medical system, Houston, TX, USA).

After treatment, mice were euthanized by decapitation and trunk blood was collected in ice-chilled heparinized tubes. Blood was centrifuged at 10,000×g for 10 minutes at 4°C. Plasma was separated, aliquoted and stored at −80°C. Tissues were removed from mice, frozen in liquid nitrogen, and stored at −80°C. All experimental procedures were approved by the Wright State University Animal Care and Use Committee.

### Blood Glucose Levels and Glucose Tolerance Test

FreeStyle® Blood Glucose Test Strips and FreeStyle Lite® Blood Glucose Monitoring System (Abbott, CA, USA) were used to determine whole blood glucose levels on a drop of tail blood collected between 10 am and midday, or at intervals thereafter as indicated. For glucose tolerance test, mice were fasted overnight for 16 h and injected with glucose (1.5 mg/kg, I.P.). Blood samples (5 µl) were collected in heparinized capillary tubes from the tip of the tail at 0, 15, 30, 45, 60, 90, and 120 min after glucose injection and diluted in 10% Lysis M Reagent (Roche Diagnostics, IN, USA). Glucose concentration was determined colorimetrically using a glucose oxidase/peroxidase reagent kit (Sigma, MO, USA). Samples were incubated with 100 µl of assay reagent at 37°C for 30 min. The reaction was stopped by adding 12 N H_2_SO_4_ and measured at 540 nm using a Fusion Packard plate reader. Values were expressed in mg/dL. Area under the glucose tolerance curve (0–120 min) was carried out using Prism software.

### Urine Collection

Mice were placed individually in metabolic cages for 24-hour urine collection with free access to food and water. Urine specimens were collected every 12 hour and kept at 4°C until the 24-hour collection period was completed. To prevent protein degradation, a total of 10 µl of protease inhibitor (Roche Diagnostics, IN, USA) was added to the collection tubes during the 24-hour collection period. Urine samples were centrifuged at 10,000×g for 5 min at 4°C to remove debris and supernatants were aliquoted and stored at −80°C for later use.

### Urinary Albumin, Creatinine and Glucose Excretion Assay

Quantitative urinary albumin was measured using a mouse ELISA kit purchased from Bethyl Laboratories (Montgomery, TX, USA). Urinary glucose excretion was measured in 2 µl urine samples using a glucose oxidase/peroxidase reagent kit as described above. Urinary creatinine was measured in 2 µl urine samples using a kit purchased from Quidel (San Diego, CA, USA). Final absorbance was read at 450 nm in a Fusion Packard plate reader.

### Kidney Histology and Immunofluorescence

Mice were anaesthetized and perfused transcardially with ice cold PBS and 10% formalin. Perfused kidneys were dehydrated, embedded in paraffin, sectioned at 4 µm thickness and mounted on glass slides. Paraffin sections were stained with periodic acid-Schiff (PAS) and Masson’s trichrome and examined under light microscopy.

For immunofluorescence, kidney sections were incubated with goat anti-ACE2 (1∶150, R&D Systems, MN, USA), rabbit anti-ADAM17 (1∶200, Enzo Life Sciences, NY, USA) or goat anti-nephrin (1∶200, R&D Systems) primary antibody followed by incubation with donkey anti-goat or anti-rabbit Cy3-conjugated secondary antibody (1∶100, Jackson ImmunoResearch, PA, USA). For double staining, donkey anti-goat Fluorescein-conjugated secondary antibody (1∶100, Jackson ImmunoResearch, PA, USA) was used. Images were captured using a FV1000 Confocal Microscope (Olympus, PA, USA). MetaMorph software (Molecular Devices, CA, USA) was used for quantitation.

### Western Blot

Renal and urinary protein expressions were determined using western blot analysis Kidneys were homogenized in Complete Lysis-M EDTA-free buffer (Roche Applied Science, IN, USA) containing 2.5 mmol/L PMSF. Kidney lysates (50 µg protein) or urine samples (10–20 µl) adjusted to creatinine were separated on a 10% SDS-PAGE gel and electroblotted to PVDF membranes (Millipore, MA, USA). After blocking for 1 hour, the membranes were incubated with goat anti-mouse ACE2 (1∶1000), rabbit anti-ADAM17 (1∶500) or goat anti-TIMP3 (1∶200, Santa Cruz Biotechnology) primary antibody overnight at 4°C followed by incubation with HRP-conjugated donkey anti-goat (1∶2000 R&D Systems, USA) or donkey anti-rabbit (1∶20000, Jackson ImmunoResearch) secondary antibody. Signals were detected using supersignal chemiluminescent substrate (Thermo Scientific, IL, USA) and visualized with a Fujifilm image analyzer (LAS 3000, Image Quant, CA, USA).

### ACE Activity

ACE activity was measured using an assay kit purchased from ALPCO Diagnostics Ltd. Briefly, 10 µl plasma was incubated with 100 µl of HEPES buffer (pH 8.0) containing the synthetic substrate [^3^H]-hippuryl glycine glycine ([^3^H]-Hip-Gly-Gly) at 37°C. After 60 min incubation, the reaction was terminated by adding 50 µl of 1 N hydrochloric acid. Liberated [^3^H]-hippuric acid, due to ACE activity in samples, was separated from unreacted substrate by addition of 1.5 ml of scintillating fluid and measured in a β-counter. The ACE activity is expressed as units/L.

### ACE2 Activity Using Fluorometry

Urinary, renal and plasma ACE2 activity was measured using the synthetic fluorogenic substrate, Mca-APK (Dnp) (Biomol International, NY, USA), with some modifications. Samples (30–40 µg protein) were incubated with 100 µl of the reaction buffer (50 mM Tris, 5 mM ZnCl_2,_ 150 mM NaCl_2_ and 10 µM lisinopril) and 50 µM Mca-APK (Dnp) for 0.5–2 h at 37°C. Fluorescence was measured at an excitation of 328 nm and emission of 393 nm using a Fusion Packard plate reader.

### ACE2 Activity Using Mass Spectrometry

To further confirm the presence of ACE2 activity in urine, matrix assisted laser desorption/ionization (MALDI) mass spectrometry was used as described before with some modifications [34]. Urine (2 µl) was incubated for 1.5 h at 37°C in 50 mM MES buffer pH 6.75 containing 0.5 µM Ang II, 2 mM PMSF and 20 µM bestatin. The reaction was stopped by acidification with trifluoroacetic acid (TFA, final concentration 1%). Peptides were purified using a C18 Ziptip (Millipore, MA, USA). Mass spectra were obtained using an Autoflex III smartbeam MALDI time-of-flight (TOF)/TOF instrument (Bruker Daltonics, MA, USA) operated with positive polarity in reflectron mode. A total of 3000 laser shots were acquired randomly for each spot in the range of *m/z* 500–3000 at a laser frequency of 100 Hz. Spectra were mass calibrated using a Bruker peptide calibration standard II.

### Plasma Hormone and Lipids Measurement

Samples were measured at the Mouse Metabolic Phenotyping Centre (Cincinnati, OH, USA). Plasma triglycerides were measured using commercially available assay kit (Randox Laboratories, UK). Plasma adiponectin concentration was measured with a mouse adiponectin ELISA kit (Millipore, St. Charles, MI, USA). Plasma levels of insulin and glucagon were measured using the Milliplex**®** MAP mouse metabolic hormone magnetic bead panel. Absorbance was measured using Luminex 200 (Millipore, Austin, TX). Plasma levels were calculated using standards provided with the Luminex kit.

### Statistics

Statistical analysis was performed using GraphPad Prism and Statistica software. All data were expressed as mean ± SEM. Unpaired student’s *t*-*t*est was used to evaluate the differences between two groups. For more than two groups, one-way ANOVA was used. The difference in blood glucose was assessed by repeated measures two-way ANOVA followed by Bonferroni’s multiple comparison test. A value of *p*<0.05 was considered statistically significant.

## Results

### Rosiglitazone Normalizes Hyperglycemia and Improves Glucose Handling in *db/db* Mice

**Figure 1:**
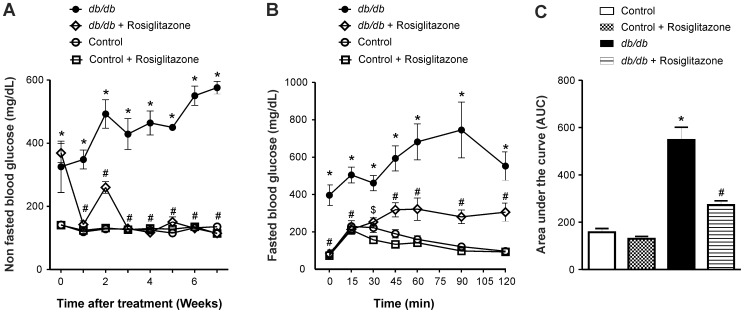
Chronic treatment with rosiglitazone (10–20 mg/kg/day) normalized hyperglycemia and improved glucose tolerance in *db*/*db* mice. (A) Non fasted blood glucose levels in control, control+rosiglitazone, *db/db* and *db/db*+rosiglitazone mice. Repeated measures two-way ANOVA using a Bonferroni’s posthoc test showed that treatment with rosiglitazone caused a significant decrease in blood glucose levels of *db*/*db* mice [F (3, 54) = 176.04], *p*<0.0001. Similarly, duration of treatment showed a significant decrease in blood glucose levels of *db/db* mice [F (21, 54) = 16.34], *p*<0.0001. Data are represented as mean ± SEM of group size (n = 6–8). (B) Glucose tolerance test in rosiglitazone treated and untreated lean control and *db*/*db* mice. After eight weeks of treatment with rosiglitazone, mice were fasted for 16 hours and dosed with glucose (1.5 g/kg I.P). Blood glucose levels were measured by tail tip bleed at 0, 15, 30, 60, 90 and 120 minutes post administration. *p<0.001 Vs age-matched lean control and lean control+rosiglitazone mice. ^#^ *p*<0.001, ^$^ *p*<0.01 Vs untreated *db/db* mice. Data are represented as mean ± SEM of group size (n = 6–8). (C) One-way ANOVA of area under curve showed that rosiglitazone significantly improved the glucose tolerance in *db/db*+rosiglitazone mice compared to untreated *db/db* mice. *p<0.001 Vs age-matched lean control mice. ^#^ *p*<0.05 Vs untreated *db/db* mice. Each bar represents mean ± SEM of group size (n = 6–8).

Compared to control mice, six-weeks old *db/db* diabetic mice exhibited significantly higher blood glucose levels, which consistently increased throughout the study period (Figure 1a). As shown in Figure 1a, chronic treatment of *db/db* mice with rosiglitazone significantly decreased blood glucose after one week and normalized hyperglycemia throughout the eight-week duration of treatment. Using an intra-peritoneal glucose tolerance test, *db/db* mice showed impaired glucose utilization compared to age-matched control mice, which was significantly improved in rosiglitazone-treated *db/db* mice (Figure 1b, c). These effects were not observed in lean control mice treated with rosiglitazone.

### Treatment with Rosiglitazone Affects General Metabolic Parameters in *db/db* Mice

As summarized in Table 1, body weight, absolute body fat, food intake, water intake, and urine volume of *db/db* mice consistently increased with age compared to control mice and control mice treated with rosiglitazone. In addition, plasma insulin, glucagon, triglyceride and glucose levels were significantly increased in *db/db* mice compared to control mice and control mice treated with rosiglitazone, while plasma adiponectin significantly decreased (*p*<0.05). Chronic treatment of *db/db* mice with rosiglitazone demonstrated a significant increase in body weight and absolute body fat compared to untreated *db/db* mice, but food intake was not different. Furthermore, rosiglitazone treatment of *db/db* mice resulted in a significant decrease in water intake and urine volume, similar to levels observed in control mice. In contrast, treatment of *db/db* mice with rosiglitazone increased plasma adiponectin levels and decreased glucagon, triglyceride and glucose levels, but had no effect on plasma insulin levels.

**Table 1:** Age dependent changes in general metabolic parameters of control, control+rosiglitazone, *db/db* and *db/db*+rosiglitazone mice.

Mice strain	Control	Control Rosi	*db/db*	*db/db* Rosi	Control	Control Rosi	*db/db*	*db/db* Rosi	Control	Control Rosi	*db/db*	*db/db* Rosi
Age (wks)	6	6	6	6	10	10	10	10	14	14	14	14
Duration ofTreatment (wks)	0	0	0	0	4	4	4	4	8	8	8	8
Body weight (g)	22.1±0.7	21.6±0.6	32.2±1.1*^,$^	30.6±1.2*^,$^	25.1±0.5	27.8±0.7	40.9±0.7*^,$^	54±0.9*^,#,$^	27.1±0.4	30.4±0.8	40.3±0.6*^,$^	63.1±0.6*^,#,$^
Absolute bodyfat (g)	ND	ND	ND	ND	4.2±0.3	ND	22.0±0.7*	33.1±0.9*^,#^	5.1±0.5^$^	10.3±0.8*	21.3±0.4*^,$^	39.5±0.6*^,#,$^
Food intake^a^(g/day)	3.4±0.2	3.5±0.3	6.3±0.8*^,$^	5.8±0.4*^,$^	3.8±0.2	4.5±0.8	7.9±0.4*^,$^	7.2±0.8*^,$^	3.5±0.1^$^	4.6±0.2*	6.8±0.8*^,$^	5.9±0.2*^,$^
Water intake(ml/day)	8.1±0.2	8.2±0.4	15.4±3.1*	12.5±0.6*^,$^	8.8±0.6	8.6±0.4	30.7±2.1*	11.6±0.3*^,#,$^	6.4±0.3^$^	9.1±0.5*	31.4±3.7*^,$^	6.5±0.2^#,$^
Urine volume(ml/day)	1.0±0.004	ND	2.6±0.2*	ND	0.8±0.1	ND	12.6±1.6*	1.2±0.2^#^	0.9±0.01	ND	23.6±1.2*	0.5±0.1^#^
Plasma insulin(ng/mL)	ND	ND	ND	ND	ND	ND	ND	ND	1.7±0.2	1.6±0.2	5.9±1.7*^,$^	4.3±0.4*^$^
Plasmaadiponectin(µg/mL)	ND	ND	ND	ND	ND	ND	ND	ND	2.0±0.1^$^	5.5±0.4	1.4±0.1*^,$^	9.1±1.5*^,#,$^
Plasmaglucagon(pg/mL)	ND	ND	ND	ND	ND	ND	ND	ND	76.0±7.2	69.0±5.7	401.4±16.5*^,$^	214.5±9.2*^,#,$^
Plasmatriglycerides(mg/dL)	ND	ND	ND	ND	ND	ND	ND	ND	94.5±5.1	86.8±5.9	274.0±24.7*^,$^	65.9±6.7*^,#,$^
Plasma glucose (mg/dL)	ND	ND	ND	ND	ND	ND	ND	ND	172.5±5.0	165.6±9.1	677.6±73.1*^,$^	158.8±27.7^#^

Values represent mean ± SEM. *p<0.05 Vs age-matched control mice, ^$^ *p*<0.05 Vs. age-matched control+rosiglitazone mice and ^#^ *p*<0.05 Vs age-matched *db/db* mice were considered statistically significant. ND means not determined. ^a^ The amount of food spilled was minimal and was not accounted for in the data presented in the table.

### Rosiglitazone Decreases Glucose and Albumin Excretion in *db/db* Mice

**Figure 2:**
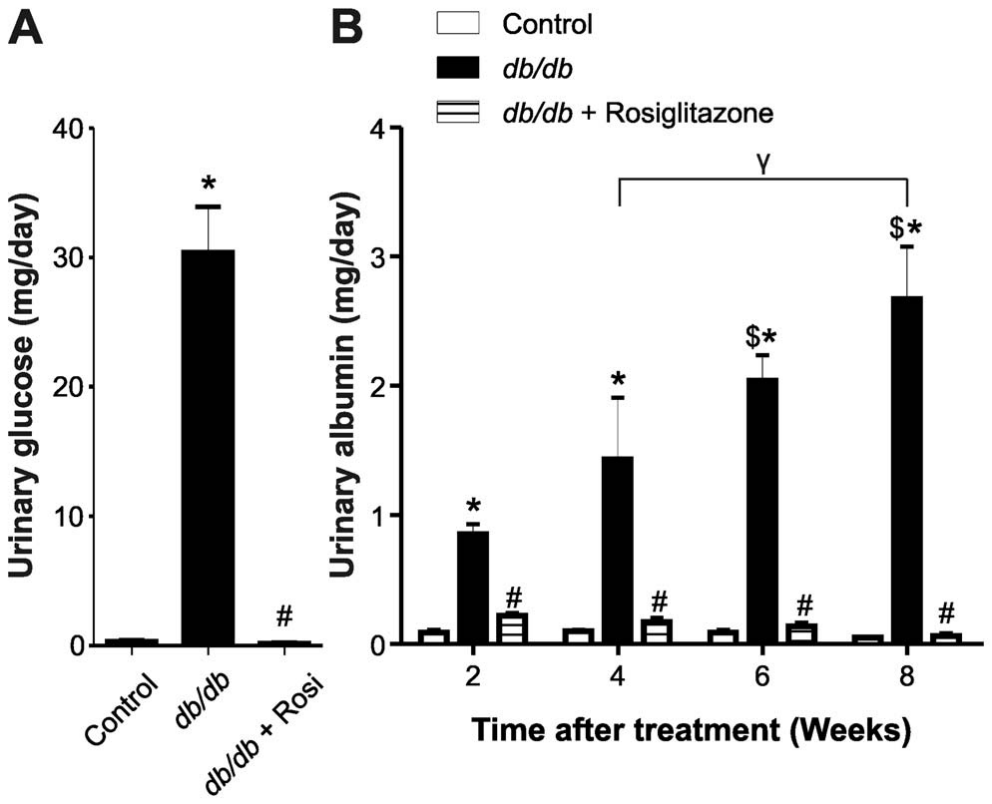
Chronic treatment with rosiglitazone attenuated glucose excretion, albuminuria and plasma creatinine levels in *db/db* mice. (A) Urinary glucose excretion in lean control, *db/db* and *db/db+*rosiglitazone mice. One-way ANOVA showed that urinary glucose excretion increased in *db/db* mice compared to lean controls (*p<0.001). Eight weeks after treatment commenced there was a significant decrease in urinary glucose excretion of *db/db+*rosiglitazone mice compared to untreated *db/db* mice. #*p*<0.001 Vs untreated *db/db* mice. Each bar represents mean ± SEM of group size (n = 6–8). (B) Urinary albumin excretion in control, rosiglitazone treated and untreated *db/db* mice 2 wks, 4 wks, 6 wks and 8 wks after the commencement of treatment. Repeated measures two-way ANOVA using a Bonferroni’s posthoc test showed that treatment resulted in a significant decrease in urinary albumin excretion of *db/db*+rosiglitazone mice [F (1, 20) = 36.004], *p*<0.0001. Similarly, duration of treatment showed a significant decrease in urinary albumin excretion of *db/db*+rosiglitazone mice after 2 wks, 4 wks, 6 wks and 8 wks of treatment [F (2, 20) = 7.70], *p*<0.001. *p<0.05 Vs age-matched lean control mice. ^#^ *p*<0.001 Vs untreated *db/db* mice. ^$^ *p*<0.05 Vs 2 wks untreated *db/db* mice. ^γ^ *p*<0.05 4 wks Vs 8 wks untreated *db/db* mice. Each bar represents mean ± SEM of group size (n = 6–7).

There was a significantly higher urinary glucose excretion in *db/db* mice compared to control mice. Chronic treatment of *db/db* mice with rosiglitazone significantly decreased urinary glucose excretion compared to untreated *db/db* mice (*p*<0.001, Figure 2a). Figure 2b illustrates a significant difference in the urinary albumin excretion between *db/db* mice and control mice. Progression of the disease led to worsening of albuminuria. Chronic treatment with rosiglitazone significantly ameliorated urinary albumin excretion in *db/db* mice after only two weeks of treatment (*p*<0.001). This reduction of urinary albumin was maintained throughout the duration of the study.

### Rosiglitazone Attenuates Renal Injury in *db/db* Mice

**Figure 3:**
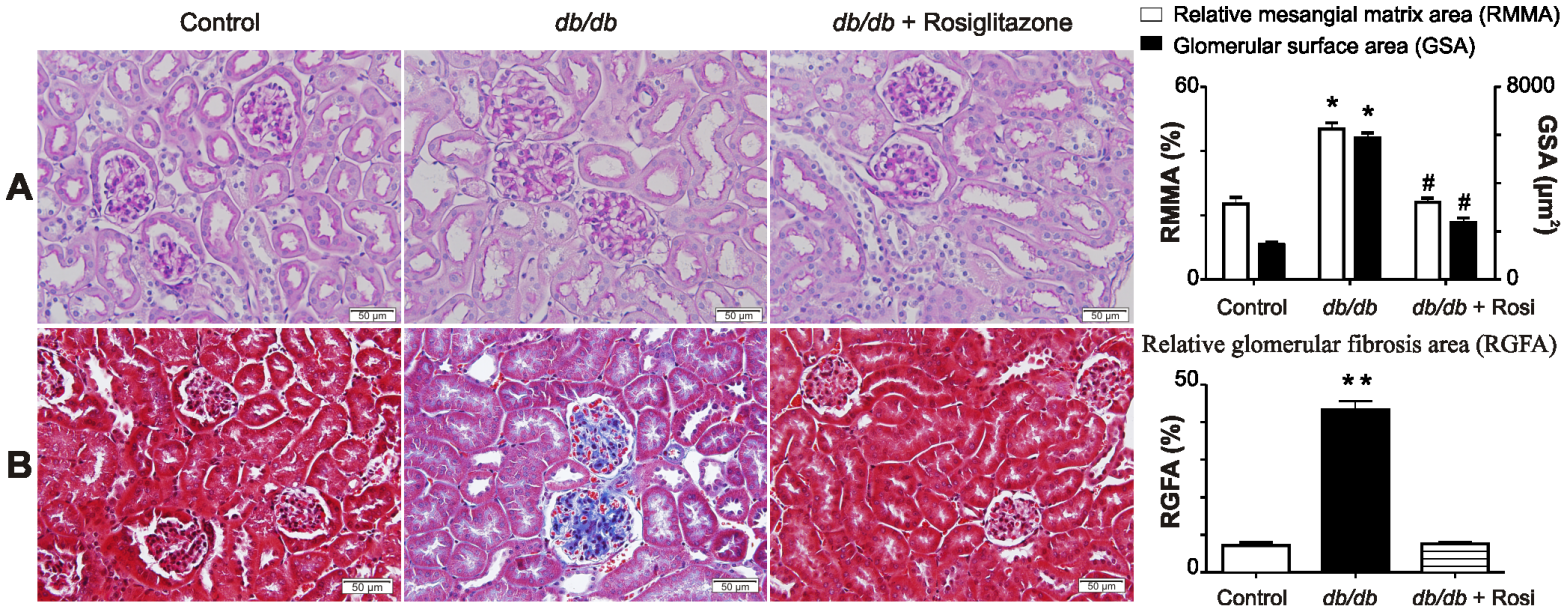
Histological analysis using PAS and Masson’s Trichrome stain after 8 weeks of treatment with rosiglitazone. (A) Representative photomicrographs depicting PAS staining of kidney sections in control, untreated and rosiglitazone treated *db/db* mice. Mesangial matrix and glomerular surface areas were significantly increased in *db/db* mice compared to control mice. Eight weeks after treatment with rosiglitazone there was a significant decrease in relative mesangial matrix area as well as glomerular surface area compared to untreated *db/db* mice. *p<0.001 Vs control mice.^ #^ *p*<0.001 Vs untreated *db/db* mice. Each bar represents mean ± SEM of group size (n = 6–12). (B) Representative photomicrographs depicting Masson’s Trichrome staining of kidney sections in control, untreated and rosiglitazone treated *db/db* mice. Glomerular fibrotic areas were significantly increased in *db/db* mice compared to control mice. Eight weeks after treatment with rosiglitazone there was a significant decrease in glomerular fibrotic area compared to untreated *db/db* mice. **p<0.0001 Vs control mice. Each bar represents mean ± SEM of group size (n = 10–11).

Glomerular tufts of *db/db* mice revealed a significant increase in mesangial expansion and surface area, which was significantly reduced after treatment with rosiglitazone (*p*<0.001, Figure 3a). In addition, renal fibrosis in *db/db* mice was significantly decreased following treatment with rosiglitazone (*p*<0.001, Figure 3b).

### Rosiglitazone Increases Nephrin Expression but Decreases Renal ADAM17 Expression and Urinary ACE2 Protein Excretion in *db/db* Mice

**Figure 4:**
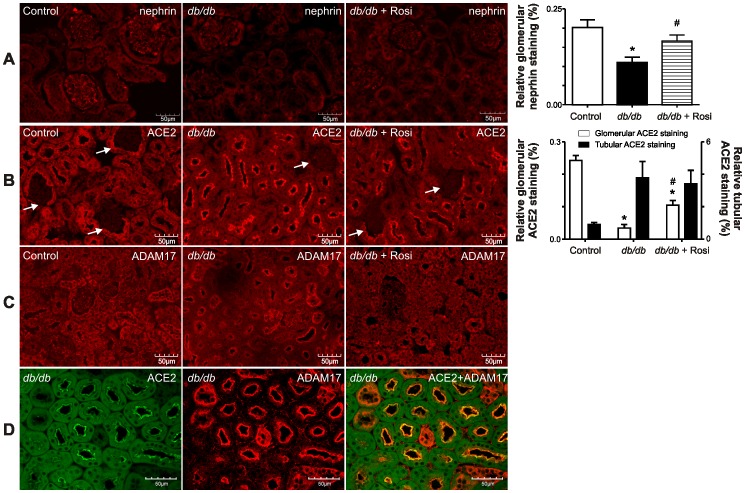
Immunofluorescence of nephrin, ACE2 and ADAM17 after 8 weeks of treatment with rosiglitazone. (A) Immunofluorescence staining for nephrin in the glomeruli of control, untreated and rosiglitazone treated *db/db* mice at 20× magnification. Nephrin expression was significantly decreased in *db/db* mice. After eight weeks of treatment with rosiglitazone there was a significant increase in nephrin expression compared to untreated *db/db* mice. *p<0.01 Vs control mice.^ #^ *p*<0.05 Vs untreated *db/db* mice. Each bar represents mean ± SEM of group size (n = 11–18). (B) Immunofluorescence staining for ACE2 in cortical tubules and glomeruli of control, untreated and rosiglitazone treated *db/db* mice at 20× magnification. White arrows indicate glomeruli. While tubular ACE2 expression was increased, glomerular ACE2 expression was significantly decreased in *db/db* mice. After eight weeks of treatment with rosiglitazone there was a significant increase in glomerular ACE2 expression while tubular ACE2 expression was unchanged compared to untreated *db/db* mice. *p<0.001 Vs control mice.^ #^ *p*<0.01 Vs untreated *db/db* mice. Each bar represents mean ± SEM of group size (n = 11–18). (C) Immunofluorescence staining for ADAM17 in cortical tubules of control, untreated and rosiglitazone treated *db/db* mice at 20× magnification. (D) Immunofluorescence double staining for ACE2 and ADAM17 in cortical tubules of *db/db* mice at 60× magnification.

**Figure 5:**
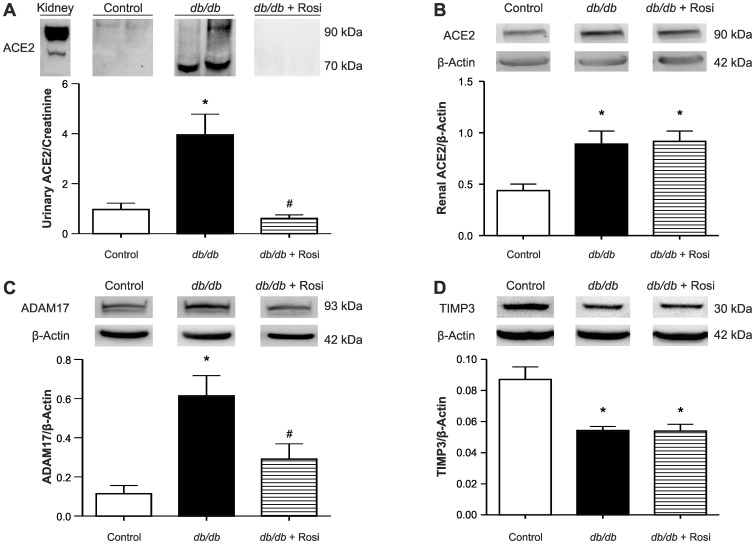
Western blot analysis for ACE2 protein expression in urine and ACE2, ADAM17 and TIMP3 protein expression in the kidney of *db/db*, *db/db*+rosiglitazone mice. (A) Western blot analysis for ACE2 protein expression in urine. Lane 1 represents mouse kidney lysate (positive control), lane 2 and 3 represent urine samples from 6 wks old control mice, lanes 4 and 5 represent urine samples from 6 wks old *db/db* mice, lanes 6 and 7 represent urine samples from 7 wks old *db/db*+rosiglitazone mice. Immunoreactive band for ACE2 was observed at 90 kDa in mouse kidney lysate (lane 1), but was seen at ∼70 kDa in the urine (lanes 4 and 5). One-way ANOVA showed that urinary ACE2 protein excretion significantly increased in *db/db* mice compared to their age-matched lean control mice. Rosiglitazone treatment decreased urinary ACE2 protein excretion compared to untreated *db/db* mice. *p<0.01 Vs age-matched lean control mice. ^#^ *p*<0.05 Vs untreated *db/db* mice. Each bar represents mean ± SEM of group size (n = 6–8). (B) Western blot analysis for ACE2 protein expression in kidney. One-way ANOVA showed that ACE2 protein expression was significantly increased in *db/db* mice compared to their age-matched lean control mice. There was no significant difference in the ACE2 protein expression in the treated *db/db* mice compared to untreated *db/db* mice after eight weeks of treatment. Each bar represents mean ± SEM of group size (n = 4–5). (C) Western blot analysis for renal ADAM17 protein expression. One-way ANOVA showed that renal ADAM17 significantly increased in *db/db* mice compared to their age-matched lean control mice. ADAM17 protein expression was significantly decreased in treated *db/db* mice compared to untreated *db/db* mice after eight weeks of treatment. *p<0.05 Vs age-matched lean control mice. Each bar represents mean ± SEM of group size (n = 3–5). (D) Western blot analysis for renal TIMP3 protein expression. One-way ANOVA showed that renal TIMP3 significantly decreased in untreated and treated *db/db* mice compared to 30 wks old lean control mice. *p<0.01 Vs lean control mice. Each bar represents mean ± SEM of group size (n = 6).

Immunofluorescence showed that protein expression of nephrin and glomerular ACE2 was decreased in the kidneys of *db/db* mice, while tubular ACE2 and ADAM17 were increased (Figure 4). These expression profiles were reversed for glomerular ACE2, nephrin and ADAM17 in rosiglitazone treated *db/db* mice, with no effect on tubular ACE2. Western blot analysis detected a strong immunoreactive band for ACE2 at ∼70 kDa in the urine of *db/db* mice (Figure 5a). Excreted ACE2 levels were significantly higher in *db*/*db* mice relative to control mice or treated *db/db* mice. Similarly, renal ACE2 protein expression (∼90 kDa) was increased in untreated *db/db* mice compared with control mice (Figure 5b). However, there was no significant difference in the renal ACE2 protein expression of rosiglitazone treated and untreated *db/db* mice (Figure 5b). Expression of renal ADAM17 followed an expression profile identical to urinary ACE2 levels with an increase in *db/db* mice, which was reversed by rosiglitazone treatment (Figure 5c). Renal TIMP3 protein expression was significantly decreased in untreated and treated *db/db* mice, as compared with control mice (Figure 5d).

### Rosiglitazone Modulates ACE and ACE2 Activities in Urine, Plasma and Kidney of *db/db* Mice

**Figure 6:**
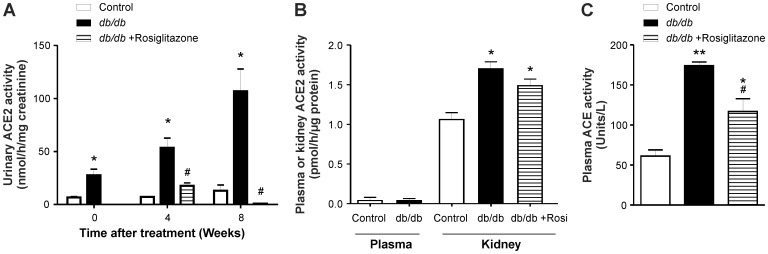
ACE2 and ACE activity in urine, plasma and kidney of control, *db/db* and *db/db*+rosiglitazone mice using a fluorometric enzyme assay. (A) Urinary ACE2 activity in control, *db/db* and *db/db*+rosiglitazone mice before and after the commencement of treatment. Two-way ANOVA showed an increase in urinary ACE2 activity of *db/db* mice compared to control mice. Four and eight weeks after treatment commenced there was a significant decrease in urinary ACE2 activity of the *db/db*+rosiglitazone mice compared to untreated *db/db* mice. *p<0.001 Vs control mice.^ #^ *p*<0.001 Vs untreated *db/db* mice. Each bar represents mean ± SEM of group size (n = 6–7). (B) Plasma and renal ACE2 activity in control, *db/db* and *db/db+*rosiglitazone mice. There was no plasma ACE2 activity in control and *db/db* mice but a significant increase in renal ACE2 activity of *db/db* mice compared to control mice was observed. Treatment with rosiglitazone had no significant effect on renal ACE2 activity of treated *db/db* mice compared to untreated *db/db* mice.*p<0.05 Vs control kidney. Each bar represents mean ± SEM of group size (n = 5–8). (C) Plasma ACE activity in control, *db/db* and *db/db*+rosiglitazone mice 8 wks after the commencement of treatment. One-way ANOVA showed an increase in plasma ACE activity of *db/db* mice compared to control mice. Eight weeks after treatment commenced there was a significant decrease in plasma ACE activity of the *db/db*+rosiglitazone mice compared to untreated *db/db* mice. *p<0.05, **p<0.001 Vs control mice.^ #^ *p*<0.05 Vs untreated *db/db* mice. Each bar represents mean ± SEM of group size (n = 6–7).

There was a significant increase in urinary and renal ACE2 activity of *db/db* mice compared to lean control mice (Figure 6a, b, *p*<0.05). Chronic treatment with rosiglitazone significantly reduced urinary ACE2 activity in treated *db/db* mice compared to untreated *db/db* mice (Figure 6a). However, treatment with rosiglitazone had no significant effect on renal ACE2 activity in treated *db/db* mice compared with untreated *db/db* mice (Figure 6b). Quantitative estimation of ACE2 enzyme activity showed that there was no detectable plasma ACE2 activity in control or *db/db* mice. In contrast, ACE activity was detectable in plasma and markedly increased in untreated and treated *db/db* mice (Figure 6c).

**Figure 7:**
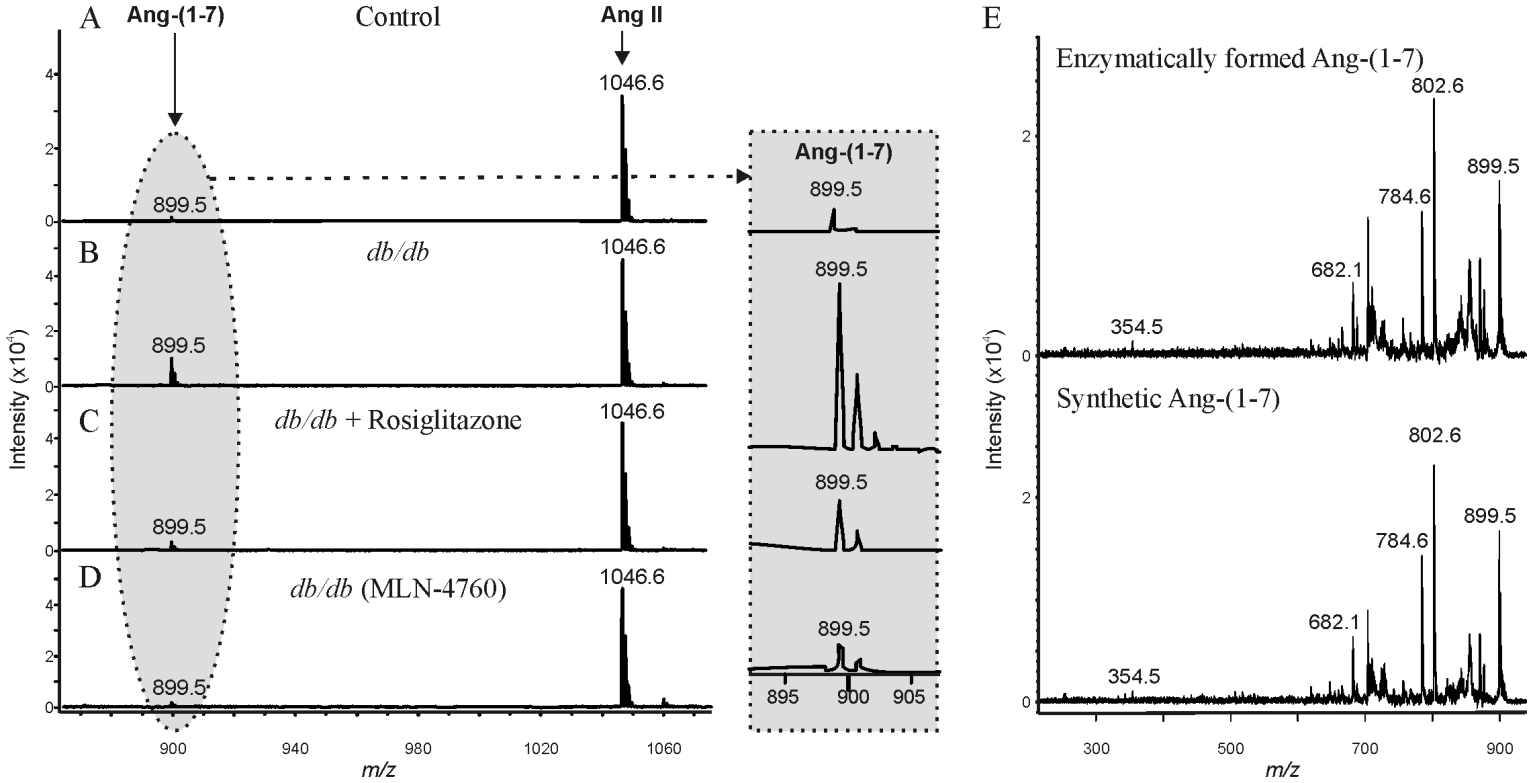
Mass spectrometric analysis of ACE2 activity in urine from control, *db/db* and *db/db*+rosiglitazone mice. Urine (2 µl) was incubated for 1.5 h at 37°C in 50 mM MES buffer pH 6.75 containing 0.5 µM Ang II, 2 mM PMSF and 20 µM bestatin. Shown is the conversion of Ang II (*m/z* 1046) to Ang-(1–7) (*m/z* 899). (A) Urinary ACE2 activity in control mice. (B) Urinary ACE2 activity in *db/db* mice. (C) Urinary ACE2 activity in *db/db* mice treated with rosiglitazone. (D) Urinary ACE2 activity in *db/db* mice in incubations with the ACE2 inhibitor, MLN-4760. (E) MS/MS of enzymatically generated Ang-(1–7) (upper panel) and synthetic Ang-(1–7) (lower panel).

The presence of urinary ACE2 activity was confirmed using a sensitive MALDI MS approach. Urine samples were incubated with the natural ACE2 substrate, Ang II, and the formation of Ang-(1–7) was used as index of ACE2 activity. Urinary Ang-(1–7) formation in *db/db* mice was elevated compared to controls and treatment with rosiglitazone restored ACE2 activity to control levels (Figures 7a–c). Formation of urinary Ang- (1–7) was attributed to ACE2 since it was reduced by 82% in the presence of specific ACE2 inhibitor, MLN-4760 (Figure 7d). The chemical nature of the generated peptide was verified by MS/MS showing that the MS/MS spectrum for Ang-(1–7) generated enzymatically in urine was identical to the MS/MS spectrum of synthetic Ang (1–7) spiked into urine reaction mixtures (Figure 7e).

### Linear Regression Analysis between Urinary ACE2 Activity and Urinary Albumin, Plasma Glucagon, Plasma Triglycerides and Blood Glucose Levels

**Figure 8:**
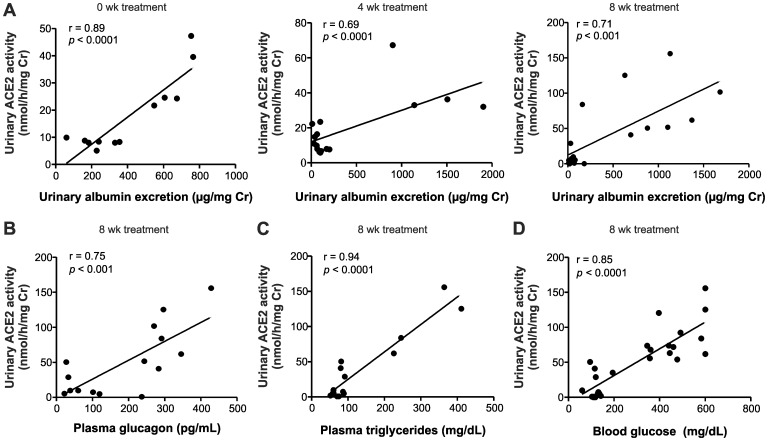
Linear regression analysis between urinary ACE2 activity and urinary albumin, plasma glucagon, plasma triglycerides and blood glucose levels. (A) Association of urinary ACE2 activity and urinary albumin excretion in control, *db/db* and *db/db*+rosiglitazone mice before and 4 or 8 weeks after commencement of rosiglitazone treatment. (B) Correlation between plasma glucagon and urinary ACE2 activity in control, *db/db* and *db/db*+rosiglitazone mice 8 weeks after commencement of rosiglitazone treatment. (C) Correlation between plasma triglycerides and urinary ACE2 activity in control, *db/db* and *db/db*+rosiglitazone mice 8 weeks after commencement of rosiglitazone treatment. (D) Correlation between urinary ACE2 activity and non fasted blood glucose levels in control, *db/db* and *db/db*+rosiglitazone mice 8 weeks after commencement of rosiglitazone treatment.

Relationships of urinary ACE2 excretion to urinary glucose excretion and to major metabolic risk factors including blood glucose, plasma glucagon and lipid concentrations were investigated in *db*/*db* diabetic mice. Linear regression analysis showed a significant positive correlation between urinary ACE2 activity and albumin excretion, plasma glucagon, plasma triglycerides and blood glucose (Figure 8).

## Discussion

This study is the first to demonstrate that shedding of renal ACE2 into urine is increased in *db/db* diabetic mice. This urinary ACE2 excretion correlated positively with the progression of diabetic renal injury represented by progressive albuminuria, mesangial matrix expansion and renal fibrosis. Overactivation of the RAS in diabetes, especially Ang II and ACE [3], leads to hypertension, fluid retention, and inflammation, causing renal and vascular end-stage disease in the long term [35]. Ang II plays a crucial role in the pathogenesis and progression of diabetic renal disease by affecting intraglomerular capillary pressure, the podocyte skeleton, and components of the slit diaphragm leading to glomerular sclerosis and microalbuminuria [36]. There is evidence that deletion of ACE2 leads to the development of Ang II dependent renal damage, suggesting ACE2 as renoprotective target in diabetes [37]. The ability of ACE2 to inactivate Ang II and generate the putative reno- and cardio-protective metabolite, Ang-(1–7), suggests that ACE2 is an important participant in cardiovascular homeostasis [38], [39]. Indeed, we found an activation of renal ACE2 in *db/db* mice, most likely a part of a mechanism to compensate for elevated Ang II levels. Moreover, our results demonstrated that ADAM17, a protease known to be involved in the ectodomain shedding of several integral proteins, including ACE2 but not ACE [40], was significantly upregulated in the kidneys of *db/db* mice. Accordingly, treatment with Ang II results in enhanced accumulation of renal ADAM17 [32]. This study is also the first report showing upregulation of renal ADAM17 in *db*/*db* mice. Moreover, renal TIMP3 expression was significantly reduced, supporting earlier findings that the loss of endogenous ADAM17 inhibitor exacerbates diabetic nephropathy [24]. Based on these data, we postulate that during early stages of kidney damage, renal ACE2 and sheddase activity of ADAM17 increase in the event of high circulating Ang II, while expression of renal TIMP3 is reduced. In consensus with our hypothesis, immunostaining results demonstrated that ADAM17 colocalized with tubular ACE2 in diabetic kidney. Consequently, due to the actions of ADAM17, proteolytically active forms of ACE2 from the kidney are shed into urine of *db/db* diabetic mice. Thus, the loss of renoprotective enzyme ACE2 could contribute to kidney damage.

One of the primary goals of managing patients with type 2 diabetes is preventing or delaying the development of diabetic renal disease [41]. Hyperglycemia leads to nephropathy by various mechanisms, such as increased endothelial cell permeability to albumin, hypertrophy and thickening of the basement membrane. We observed a decrease in a slit diaphragm associated protein, nephrin, from the glomeruli of *db/db* mice. We hypothesize that loss of nephrin from the slit pore may result in the enhanced passage of albumin into urine, which is consistent with previous studies [42]. At five weeks of age, *db/db* mice exhibited hyperglycemia, as well as microalbuminuria. Our previous studies demonstrated that blood pressure in *db/db* mice starts to rise after eleven weeks of age [3]. Therefore, we propose that kidney damage in *db/db* mice is initially triggered by hyperglycemia. With increasing age, disease progresses and kidney function deteriorates further, resulting in a significant rise in albuminuria, mesangial expansion, and renal fibrosis.

Retrospective clinical studies suggest that strict control of hyperglycemia depreciates the progression of diabetic nephropathy and cardiovascular complications [43]. To this end, we explored the effect of normalizing glycemia by rosiglitazone on kidney function, renal ACE2 shedding, ADAM17 and TIMP3 expression in *db/db* mice. We observed after only one week of treatment of *db/db* mice a significant decline in blood glucose levels to normal levels, which was associated with decreased urinary albumin and an attenuation of renal pathologies at the end of the study period. These findings clearly demonstrate the significance of glycemic control in preventing diabetic renal injury. Treatment with rosiglitazone attenuated urinary ACE2 protein excretion and activity but had no effect on renal ACE2 in treated *db/db* mice compared to untreated *db/db* mice. Unchanged levels of renoprotective tubular ACE2 might complement the positive effects of treatment with rosiglitazone in the diabetic kidney. In addition, rosiglitazone markedly attenuated renal ADAM17 in *db/db* mice. Thus, rosiglitazone treatment may impart renoprotection via attenuated shedding of ACE2, possibly through its direct influence on ADAM17. Interestingly, treatment with rosiglitazone had no effect on TIMP3 suggesting that hyperglycemia has no control over TIMP3.In addition to the conventional fluorogenic assay, urinary ACE2 activity was further verified by MS, which is known for its high sensitivity, specificity and accuracy [34], [44]. In agreement with the Western blot results, both enzymatic test systems confirmed that urinary ACE2 activity increased significantly in *db/db* mice compared to lean controls. In contrast, rosiglitazone treatment significantly attenuated urinary ACE2 activity in *db/db* mice. Moreover, plasma ACE levels were significantly reduced in treated *db/db* mice, supporting the hypothesis that the renoprotective effects of rosiglitazone could be partly mediated by its influence on the RAS. To investigate the source of ACE2 in urine, we measured ACE2 activity in plasma and kidney of *db/db* mice. ACE2 activity has previously been observed in sheep serum [45] and plasma of diabetic rodent models [15], [46]. However, plasma ACE2 activity was not detectable in normal and healthy subjects [47]. In turn, the latter attributed this effect to the presence of endogenous ACE2 inhibitor. Another study showed an elevation in serum ACE2 activity of type 1 diabetic patients with macro- and microvascular complications [48]. Reasons behind this disparity might be differences in species, type of substrate used, incubation time (up to 24 hours) or the method adopted. Concurrent with our previous findings [34], we found that there is neither detectable plasma ACE2 activity in control nor *db/db* mice, but there is significantly elevated ACE2 activity in the kidneys of *db/db* mice. In addition, clinical data from CKD and diabetic renal transplant patients [17], [18] also support our conclusion that the kidney, not plasma, is the source of urinary ACE2. Thus, urinary ACE2 could be a promising non-invasive biomarker for assessing renal function in diabetic pathologies.

Determining the level of microalbuminuria is still clinically used for the diagnosis and prognosis of diabetic nephropathy. Although blood pressure control was effective for preventing onset and progression of microalbuminuria to macroalbuminuria in type 2 diabetic patients, it did not prevent the development of end stage renal disease, as shown in the ADVANCE trial [49]. Therefore, there is a need for more sensitive and specific urinary markers to early and reliably predict kidney disease. Previous studies in humans suggested RAS components, such as urinary angiotensinogen, for the determination of kidney disease in diabetic as well as CKD patients [50], [51]. In rodent models of CKD, pathophysiological changes were associated with an increased overexpression of urinary ACE suggesting that this enzyme could be used as a biological urinary marker in CKD [52]. Moreover, ACE was found in human urine and associated with hypertension [53]. We propose that urinary ACE2 can be used as a potential marker of diabetic nephropathy. Our results show that urinary ACE2 activity is elevated in *db/db* mice compared to control mice and persistently increased throughout the study period of eight wks. Excretion of urinary albumin followed the same trend and a strong positive correlation was observed between urinary ACE2 activity and albumin excretion among the groups throughout the experiment. These finding are consistent with a recent study comparing urinary ACE2 and albumin excretion in human patients [54]. Interestingly, plasma abnormalities (glucose, glucagon and triglycerides) seen in *db/db* mice demonstrated a significant positive correlation with urinary ACE2. Correlation between urinary ACE2 excretion and well-established plasma risk factors of diabetes, such as triglycerides and glucagon, suggest urinary ACE2 as a novel surrogate marker for diabetes. Based on these results, it is tempting to hypothesize that urinary ACE2 is an independent risk factor for predicting early onset of diabetes and its related complications in high-risk patients.

In conclusion, our findings suggest that rosiglitazone imparts renoprotection at least partially by decreasing renal ACE2 shedding in *db/db* mice. In conjunction with other studies in the field of urinary biomarkers for metabolic disease, our results suggest that ACE2 is a promising urinary biomarker to detect early signs of kidney disease.
